# Have Guidelines Affected Ear, Nose, and Throat Specialists’ Diagnoses and the Prescription of Antibiotics for Acute Otitis Media?

**Published:** 2012

**Authors:** Kamran Kamrava, Maryam Jalessi, Alimohamad Asghari, Mohammad Farhadi, Alireza Ahmadvand, Babak Ghalehbaghi, Mehdi Saffari jourshari, Mir Abolfazl Motiei jouibari

**Affiliations:** 1 *Assistant professor of otolaryngology, ENT, Head & Neck Department and Research Center, Hazrat Rasoul Akram Hospital, Tehran University of medical sciences*; 2 *Professor of otolaryngology, ENT, Head & Neck Department and Research Center, Hazrat Rasoul Akram Hospital, Tehran University of medical sciences*; 3 *Assistant professor in community medicine, Department of community medicine, Faculty of medicine, Tehran University of Medical Sciences*; 4 *Fellow of Allergy and Clinical Immunology, Hazrat Rasoul Akram Hospital, Tehran University of medical sciences*; 5 *Student of medical sciences, faculty of medicine, Tehran University of medical sciences*

**Keywords:** Acute otitis media, Attitude, Knowledge, Otolaryngologist

## Abstract

**Introduction:**

The Ministry of Health and Medical Education of Iran, and similar institutions in many other countries, advises physicians to use current guidelines for the diagnosis and treatment of acute otitis media (AOM). However, there has been no evaluation of the effectiveness of such guidelines or whether physicians in Iran adhere to them. Thus, as laryngologists are the most important group of people who interact with patients with AOM, the aim of this study was to evaluate the attitude of laryngologists to the established guidelines.

**Materials and Methods:**

A total of 120 anonymous surveys were mailed to 120 otolaryngologists in Tehran, Iran, to evaluate the patterns of diagnosis and treatment of AOM used by these physicians. The survey included questions regarding the otolaryngologists’ age, gender, place of work, and attitude towards diagnosis and treatment of AOM.

**Results:**

Sixty-two completed surveys were received, for a response rate of 51%. There was no significant difference between respondents to these surveys according to sex, age, practice setting, graduation year, or the number of patients with AOM seen each month.

**Conclusion:**

Our study adds new insights to the previous literature on the use of guidelines in the management of AOM. We can now assess the impact of guidelines on the usual practice of physicians in evidenced-based management of AOM.

## Introduction

Acute otitis media (AOM) is defined as an acute onset of middle-ear effusion, with signs and symptoms of middle-ear inflammation (1). It is one of the most common diseases in general practice, especially in children, with an incidence of 200 cases per 1000 patient-years for children aged 0 to 4 years (2). Antibiotics are a common treatment for AOM in many countries (3), in spite of evidence that they only have a limited effect (4, 5). The proportion of patients with AOM who receive antibiotics varies among countries, ranging from approximately 95% in the USA, Canada, UK, New Zealand, Sweden, and Spain, to approximately 50% in the Netherlands (3, 6, 7-10).

Many practice guidelines have been developed and distributed to help physicians with the clinical diagnosis and treatment of AOM. Based on these guidelines, the concept of initial observation without antibiotic treatment has been adopted as standard practice in some parts of the world (11), and is likely to achieve acceptance in the United States as well (11,12). Alongside that, the widespread emergence of antimicrobial resistance has increased the urgency to reduce antibiotic use and has made the decision to use antibiotics more difficult. In May 2004, in response to these issues, the American Academy of Pediatrics (AAP) and the American Academy of Family Physicians (AAFP) together issued clinical practice guidelines entitled “Diagnosis and Management of Acute Otitis Media” (13). Since then, the Ministry of Health and Medical Education of Iran has recommended that ear, nose, and throat (ENT) specialists adopt these guidelines for the diagnosis and treatment of AOM, but there have not been any studies to investigate the effects of the guidelines. Thus, the aim of this study was to investigate the effect of the AAP/AAFP guidelines on the diagnosis and treatment of AOM by ENT specialists in Iran. 

***Materials and Methods***

 Between February 2009 and March 2010, a total of 120 anonymous surveys were mailed to 120 ENT specialists in Tehran, Iran, to evaluate the pattern of diagnosis and treatment of AOM used by these physicians. The survey included questions on age, gender, place of work, and attitudes towards diagnosis and treatment of AOM. The survey tried to assess the knowledge and practices of physicians regarding the management of AOM cases based on medical histories and physical examination results from hypothetical patients (Table 1). These cases were developed by a panel of experts consisting of general practitioners, academic pediatricians, and otorhinolaryngologists.

In the first part of the survey, eight cases were developed and presented consecutively and physicians were asked to determine if they could diagnose AOM definitively in each case or if they were uncertain about the diagnosis. In the second part, six AOM scenarios were introduced and physicians were asked to choose between the options of “observation” and “antibiotic prescription” in any of the situations where the presented case was a “definite” or “probable” patient with AOM. In the third part of the survey, two AOM cases, three different treatment situations, and seven proposed antibiotic regimens plus an observation option were introduced. This part was designed to assess physicians’ adherence to guidelines regarding antibiotic therapy, i.e. choosing the best time to start antibiotics and the most appropriate regimen for each patient.

Written informed consent was obtained from participants beforehand and the study protocol was approved by the Tehran University of Medical Sciences (TUMS) Ethics Committee.

***Statistical analysis***

 Data were entered into and analyzed using the Statistical Package for Social Sciences (SPSS), version 16, software. Descriptive variables were expressed as percentage, median, and range. For analysis of continuous variables, all data were analyzed initially using the Kolmogorov-Smirnov test to assess for normality. Then the independent sample t-test and Mann-Whitney U test were used for comparison of parametric variables.

**Table 1:** AOM cases and scenarios developed and presented consecutively to Otolaryngologist practitioners in a three-parts survey to assess their knowledge and practice.

**Part 1: **“certain” or “uncertain” on the diagnosis of AOM based on these scenarios		
**Case Order**	**Medical History**	**Physical Examination**
1	A 5-year-old boy with acute onset of severe otalgia	A body temperature of 38.5°C distinct redness of the Tympanic membrane (TM) Fluid in the middle ear
2	A 15-year-old girl with moderate otalgia, and a history of 39°C body temperature in recent five days	Reddened TM Effusion in the middle ear
3	A 3-month-old infant with severe restlessness from 2 days ago	Body temperature of 39.5°C Red TM on otoscopy without any fluid collection in the middle ear
4	A 25-year-old man complaining of acute onset of severe otalgia	Body temperature of 38.5°C since previous 2 days Red and bulged TM
5	A 5-month-old infant with sever restlessness and a history of body temperature of 39.5°C in the recent 12 hours His mother complains of diarrhea and rhinorhea	Red TM on otoscopy
6	A 10-month-old boy with restlessness and body temperature of 38°C since yesterday and a history of touching his right ear	A reddened and bulged TM on otoscopy
7	A restless 18-month-old girl with body temperature of 39°C from yesterday who touches her left ear during examination	An equivocal otoscopic examination
8	A 32-year-old woman with a history of upper respiratory tract infection within the last week; she complains of dull pain in her right ear	Annular redness around TM on otoscopic examination
**Part 2: **To choose between “observation” and “antibiotic prescription” options		
1	An infant younger than 6 months with fever less than 39°C	
2	An infant younger than 6 months with fever more than 39°C	
3	A 6-month- to 2-year-old child with fever less than 39°C	
4	A 6-month- to 2-year-old child with fever more than 39°C	
5	A child older than 2 years with mild otalgia and fever less than 39°C	
6	A child older than 2 years with mild otalgia and fever more than 39°C	
**Part** **3: **Adherence to guidelines regarding choosing the best time to start antibiotics and the most appropriate regimen for each patient		
Cases	1. AOM with body temperature of less than 39°C and/or mild otalgia	
	2. AOM with body temperature of more than 39°C and/or severe otalgia	
Treatment situations	1. Initiate antibiotic therapy at first visit	
	2. Observe the patient and postpone antibiotic therapy for 48-72hr after observation if symptoms did not improve	
	3. Change to an alternative antibiotic regimen after 48-72hr from administration of the first antibiotic regimen if it has not evoked a proper response	
Antibiotic regimens	Low-dose Amoxicillin (40-50 mg/kg/day)	
	High-dose Amoxicillin (80-90 mg/kg/day)	
	Low-dose Co-Amoxiclav (40-50 mg/kg/day)	
	High-dose Co-Amoxiclav (80-90 mg/kg/day)	
	Azithromycin	
	Cefuroxime	
	Ceftriaxone	
	Observation without immediate antibiotic therapy	

## Results

Among the 120 surveys sent out, 62 surveys were completed by physicians for a response rate of 51%. The demographic characteristics of the respondents are shown in Table 2.

**Table 2:** Demographic characteristics of 62 Otolaryngologist participated in the survey

Age, yr (Median [range])	42(31-80)
Sex (n [%])	
Male Female	48(78.7) 13(21.3)
**Place of practice (n[%]) Hospital**	
Clinic Office Hospital & office Office & clinic Hospital & clinic & office Unknown **Duration of practice (Median [range])**	3(4.9) 1(1.6) 17(27.9) 17(27.9) 9(14.8) 14(23) 12(19.7)

The otolaryngologists reported that they see 3 to 50 probable or certain cases of AOM each month (median = 10, mean = 15.9). The sex, age, or practice setting did not differ significantly between the ENT specialists who saw either less or more than 10 cases per month. A total of 45.8% of respondents believed that they had received proper medical education concerning AOM during medical school, and for the residency period this increased to 96.7%. Those who claimed that they had received proper education concerning AOM during medical school did not differ significantly in sex, age, practice setting, or the number of AOM patients seen each month compared to those who did not claim that they had received proper education concerning AOM.

The attitudes of otolaryngologists towards a diagnosis of AOM based on the eight cases in Part 1 of the survey are shown in Table 3. Significantly more otolaryngologists who saw more than the median number of AOM cases in each month diagnosed the third and fifth case wrong (P=0.011 and P=0.006, respectively), and those who had spent more than the median number of years working as otolaryngologists diagnosed the sixth and eighth case wrong (P=0.002, P=0.033, respectively). There were no other significant differences between the respondents.

**Table 3:** Percentage Otolaryngologist diagnosed 8 hypothetical AOM cases with certainty.

**Medical History and physical examinations of 8 hypothetical cases**	**Certain** **diagnosis**	**Probable diagnosis**
A 5-year-old boy with acute onset of severe otalgia; a body temperature of 38.5°C; distinct redness of the tympanic membrane (TM); and fluid in the middle ear	95%*	5%
A 15-year-old girl with moderate otalgia; a history of 39°C body temperature in recent five days; reddened TM; and effusion in the middle ear	28.3%*	72.7%
A 3-month-old infant with severe restlessness from 2 days ago; body temperature of 39.5°C; reddened TM on otoscopy without any fluid collection in the middle ear	42.7%	58.3%*
A 25-year-old man complaining of acute onset of severe otalgia; body temperature of 38.5°C since previous 2 days; reddened and bulged TM	90%*	10%
A 5-month-old infant with sever restlessness and a history of body temperature of 39.5°C in the recent 12 hours; his mother complains of diarrhea and rhinorhea; reddened TM on otoscopy	55%	45%*
A 10-month-old boy with restlessness; body temperature of 38°C since yesterday and a history of touching his right ear; a reddened and bulged TM on otoscopy	68.3%*	31.7%
A restless 18-month-old girl; body temperature of 39°C from yesterday; touches her left ear during examination; and an equivocal otoscopic examination	5.1%	94.9%*
A 32-year-old woman with a history of upper respiratory tract infection within the last week; complains of dull pain in her right ear; and annular redness around TM on otoscopic examination	25%	75%*

*Correct answers based on AAP/AAFP guidelines

A total of 10% of the physicians surveyed indicated that they would observe without antibiotic therapy in patients with certain AOM, while 90% would decide to prescribe antibiotics. 

The answers from physicians regarding the prescription of antibiotics in certain and probable cases of AOM in the six scenarios in Part 2 of the survey are shown in Table 4. 

**Table 4:** Percentage of physicians who decided to choose “antibiotic therapy” or “observation without antibiotic therapy” in certain and probable AOM conditions for 6 different scenarios.

**Scenarios**	**Certain diagnosis**		**Probable diagnosis**	
	Observation without antibiotic therapy	Antibiotic therapy	Observation without antibiotic therapy	Antibiotic therapy
An infant younger than 6 months with fever less than 39°C	12.1	*87.9	76.7	*23.3
An infant younger than 6 months with fever more than 39°C	3.4	*96.6	11.7	*88.3
A 6-month- to 2-year-old child with fever less than 39°C	11.9	*88.1	*76.8	23.2
A 6-month- to 2-year-old child with fever more than 39°C	1.7	*98.3	15.5	*84.5
A child older than 2 years with mild otalgia and fever less than 39°C	*43.1	56.9	*90.7	9.3
A child older than 2 years with mild otalgia and fever more than 39°C	1.8	*98.2	*12.1	87.9

* Correct answers based on AAP/AAFP guidelines

When grouping the otolaryngologists by sex, age, practice setting, graduation year, or the number of AOM patients seen each month, there were no significant differences between respondents in these six scenarios. 

In the third part of the survey, where respondents had to choose a treatment regimen for two cases in three different treatment situations, only 6.7% indicated that they would prescribe an appropriate antibiotic (Amoxicillin 80–90 mg/kg/day) as an initial treatment in the first case (fever of less than 39°C and/or mild otalgia). Of the remaining respondents, 45.0% indicated that they would choose low-dose Amoxicillin (40–50mg/kg/day), 20.0% would choose low-dose Co-Amoxiclav (40–50 mg/kg/day), and 23.3% would choose observation without immediate antibiotic therapy. For initial treatment of the second case (fever of more than 39°C and/or severe otalgia), 26.7% indicated that they would prescribe an appropriate antibiotic (Co-Amoxiclav 80–90 mg/kg/day), while 28.3% would choose high-dose Amoxicillin (80–90 mg/kg/day), 25.0% would choose low-dose Co-Amoxiclav (40–50 mg/kg/day), and 13.3% would choose low-dose Amoxicillin (40–50 mg/kg/day).

For the first case, if there wasn’t an improvement after 48 to 72h of observation only 10.2% of physicians indicated that they would prescribe an appropriate antibiotic (Amoxicillin 80–90mg/kg/day); 37.3% would choose low-dose Co-Amoxiclav (40–50 mg/kg/day), 37.3% would choose low-dose Amoxicillin (40–50 mg/kg/day), and 8.5% would choose high-dose Co-Amoxiclav (80–90 mg/kg/day). For treatment of the second case, after 48 to 72h of observation without improvement 33.9% indicated that they would prescribe an appropriate antibiotic (Co-Amoxiclav 80–90mg/kg/day); 35.6% would choose high-dose Amoxicillin (80–90 mg/kg/day), and 22.0% would choose low-dose Co-Amoxiclav (40–50 mg/kg/day). A significantly greater number of otolaryngologists who were older than the median age (42 years) would treat this case according to the AOM guidelines than those who were younger than the median age (*P*= 0.006).

For the third treatment situation, only 23.7% of respondents indicated that they would prescribe an appropriate antibiotic (Co-Amoxiclav 80–90mg/kg/day) for the first case following 48 to 72h of initial antibiotic therapy without response; 44.1% would choose low-dose Co-Amoxiclav (40–50mg/kg/day), 13.6% would choose Azithromycin, and 10.2% would choose high-dose Amoxicillin (80–90mg/kg/day). In the second case, following 48 to 72h of initial antibiotic therapy without response only 5.2% indicated that they would prescribe an appropriate antibiotic (Ceftriaxone); 31.0% would choose high-dose Co-Amoxiclav (80–90 mg/kg/day), 25.9% would choose Azithromycin, 12.1% would choose low-dose Co-Amoxiclav (40–50 mg/kg/day), 10.3% would choose high-dose Amoxicillin (80–90mg/kg/day), 8.6% would choose Cefuroxime, and 6.8% would choose a combination of two antibiotics.

## Discussion

Whether practice guidelines can change physicians’ behavior is an important issue. Most studies designed to evaluate the effectiveness of clinical practice guidelines have used surveys or responses to clinical vignettes. However, there is very little evidence that physicians’ responses to such theoretical situations predict their actual behavior in practice, and studies of actual patterns of practice compared with guideline recommendations have shown poor compliance (14-16).

AOM is one of the diseases with the highest rate of treatment with antibiotics worldwide (17). While AAP and AAFP guidelines have suggested observation therapy without any medication in certain cases, it seems that physicians show little interest in following this recommendation. This could be due to the wishes of the patients or their parents or difficulty in patient follow-up (16-19). Coco and colleagues demonstrated that 82% of patients with AOM visit pediatricians, 14% go to see family physicians, and 4% consult other physicians such as ENT specialists (20). Their study also showed that antibiotics were not prescribed in 13% of cases. Vernacchio and colleagues evaluated the approach of ENT specialists to AOM and compared it with the 2004 AAP/AAFP guidelines. The results of their survey, which had a response rate of 62.7%, showed that 83.8% of ENT specialists would choose to observe their patients. However, the observation method had only been used by physicians in 15% of their cases in the prior 3 months (18). In our study, in 78.8% of the cases described the patient would have received antibiotics. This means that 21.2% of all cases would have been managed using initial observation, which is comparable with studies from other countries; however, only 6.7% of the respondents would have chosen an appropriate antibiotic.

In a comparison of our study to the study by Vernacchio and colleagues (18), both demonstrated a significant deviation from AAP/AAFP guidelines. In cases of mild AOM, more than half of Western doctors prescribed high-dose amoxicillin and nearly one-third prescribed a standard dose. In comparison, 21.6% of the Iranian ENT specialists surveyed in this study would prescribe high-dose amoxicillin and 29.4% would prescribe a standard dose. In severe AOM, only 12.5% of American physicians gave high-dose Co-Amoxiclav to their patients; in Iran 29.9% of the physicians surveyed indicated that they would prescribe high-dose amoxicillin. Another study by Coco and colleagues, demonstrated that prescription of Co-Amoxiclav decreased, even to as low as 2% in some cases, after the introduction of the guidelines. In fact, the number of antibiotics other than amoxicillin that were prescribed in visits was too low to evaluate (18). Despite the tendency of physicians in other countries not to prescribe highly potent antibiotics, even in severe infections (18, 21), nearly 21% of the Iranian doctors surveyed indicated that they would prescribe high or low-dose Co-Amoxiclav to a patient with AOM on their first visit. Another study indicates that the patterns of practice by pediatricians, otolaryngologists, and pediatric otolaryngologists differ from the recommended guidelines according to the physician’s specialty (21). We did not identify clinically significant consistent differences between specialists and general practitioners in our study, but similar to other studies our study did show a vast deviation from the AAP/AAFP guidelines.

All of these studies, like ours, demonstrate that most physicians do not follow the AAP/AAFP guidelines properly (18-22). This could be due to different species of microorganism associated with AOM, older physicians’ preferences for prescribing other medications, or disregard for the guidelines. It is quite likely that there are some aspects of four factors; knowledge, attitude, disagreement, and behavior, that are operative in most instances where guidelines have not significantly changed physician behavior (17-23). However, the studies do show an interesting trend in that prescription of broad-spectrum antibiotics for AOM steadily increased from 1998 to 2004 but declined after the guidelines were introduced (23). 

Our study has a few limitations: first, we only asked physicians based in Tehran about their practices regarding the management of AOM and this may be different in other parts of country. Second, although we tried our best to increase the response rate we received from physicians, only half responded. The non-respondents will be the subject of our future research to be included in a more comprehensive study on guideline adherence.

## Conclusion

Our study adds new insights to previous literature on the management of AOM according to published guidelines. Going forward, we can now assess the impact of guidelines on the usual practices of otolaryngologists in evidenced-based management of AOM. The most important result from the study highlights the fact that the majority of the Iranian physicians surveyed believed that they received an acceptable education on how to manage AOM during their time in medical school. Objectives of our future research in this area will include exploration of the reasons for non-compliance to the guidelines on the part of more expert physicians. It seems that recent studies to answer these kinds of questions are somehow inconclusive. Does non-compliance result from the notion that older physicians find traditional approaches easier to follow, or does it show weaknesses in the guidelines that inhibit clinicians from applying them in their actual practice?
